# Differential expression of the inflammatory *ciita* gene may be accompanied by altered bone properties in intact sex steroid-deficient female rats

**DOI:** 10.1186/s13104-023-06543-4

**Published:** 2023-12-19

**Authors:** Vivi FH Jensen, Maria Swanberg, Maria Herlin, Fiona E McGuigan, Niklas R Jørgensen, Kristina E Akesson

**Affiliations:** 1 Department of Clinical Sciences Malmö, Clinical and Molecular Osteoporosis Research Unit, Lund University, Malmö, 214 28 Sweden; 2 Department of Orthopaedics, Skåne University Hospital, Malmö, 205 02 Sweden; 3 Department of Experimental Medical Science, Translational Neurogenetics Unit, Lund University, Lund, 221 84 Sweden; 4 Department of Clinical Biochemistry, Centre of Diagnostic Investigation, Rigshospitalet, Glostrup, 2600 Denmark; 5 Institute of Clinical Medicine, Faculty of Health and Medical Sciences, University of Copenhagen, Copenhagen, 2200 Denmark

**Keywords:** *Ciita*, Bone, Inflammation, Rat, Ovariectomy, BMD, Biomechanical testing, micro-CT

## Abstract

**Objective:**

The class II transactivator (CIITA), encoded by the *CIITA* gene, controls expression of immune response regulators, which affect bone homeostasis. Previously, we investigated a functional *CIITA* polymorphism in elderly women. Women carrying the allele associated with lower CIITA levels displayed higher bone mineral density (BMD), but also higher bone loss. The present exploratory study in a rat model sought to investigate effects of differential expression of *Ciita* on bone structural integrity and strength. Two strains DA (normal-to-high expression) and DA.VRA4 (lower expression) underwent ovariectomy (OVX) or sham-surgery at ~ 14-weeks of age (DA OVX n = 8, sham n = 4; DA.VRA4 OVX n = 10, sham n = 2). After 16-weeks, femoral BMD and bone mineral content (BMC) were measured and morphometry and biomechanical testing performed.

**Results:**

In DA.VRA4 rats, BMD/BMC, cross-sectional area and biomechanical properties were lower. *Ciita* expression was accompanied by OVX-induced changes to cross-sectional area and femoral shaft strength; DA rats had lower maximum load-to-fracture. Thus, while lower *Ciita* expression associated with lower bone mass, OVX induced changes to structural and mechanical bone properties were less pronounced.

**Conclusion:**

The data tentatively suggests association between *Ciita* expression and structural and mechanical bone properties, and a possible role in bone changes resulting from estrogen deficiency.

**Supplementary Information:**

The online version contains supplementary material available at 10.1186/s13104-023-06543-4.

## Introduction

Immune response activation produces pro-inflammatory cytokines which affect bone remodeling [1]. TNF-α and IFN-γ play a role in loss of bone induced by estrogen-deficiency, leading to osteoporosis [2–4]. An important component of the inflammation-promoting cascade are the major histocompatibility complex (MHC) class II antigens [5]. MHC gene expression is regulated by the class II transactivator [5]; thus, altered expression of the *CIITA* gene affects expression of MHC class II antigens and antigen presentation.

In ovariectomised (OVX) mice, Ciita is implicated in the cascade leading to bone loss via increased IFN-γ levels, which stimulate *Ciita* expression [2]. But intact, sexually mature female mice systemically overexpressing *Ciita* also develop an osteopenic phenotype from 12 to 24 weeks of age [6].

In humans, functional polymorphisms alter *CIITA* gene expression, hence affecting CIITA levels. Previously, we investigated *CIITA* rs3087456 polymorphism in elderly women. Women carrying the allele associated with lower CIITA levels displayed higher bone mass, but higher bone loss [7]. Moreover, in the same cohort, women with persistently elevated levels of C-reactive protein, also displayed higher bone loss [8].

To investigate the connection between bone properties and different inflammatory backgrounds associated with differences in expression of the *Ciita* gene, we performed a preliminary study using a well-characterised rat model consisting of two strains with differing expression levels of *Ciita* [9–11]. In this **exploratory** study of female rats using OVX to model estrogen-deficiency, our endpoint was to describe differences in bone structural integrity and strength phenotypes.

## Main text

### Animals and measurements

Animal care and experiments were performed in accordance with the guidelines of relevant institutional authorities and approved by the Animal Ethics Committee, Lund, Sweden.

Young adult females from two inbred rat strains were used: Dark Agouti (DA) and DA.VRA4, which are genetically identical except for different alleles in the VRA4 locus on chromosome 10 including the *Ciita* gene. This results in lower *Ciita* expression in the DA.VRA4 strain (DA has normal-to-high expression). Development of the congenic strain has been described in detail [10, 12] and the differential expression characterised in a variety of tissues [9–11].

Rats were bred at the Clinical Research Centre, Lund University, Sweden. The initial number (15 DA; 14 DA.VRA4) was the minimum ethically appropriate for this exploratory study. Based on an osteopenic phenotype at 12 weeks in mice [6], rats at ~ 14wks (12-17wks) underwent bi-lateral ovariectomy (OVX) (n = 10/strain) [13] or sham-surgery (n = 4–5/strain); designated Day 0. Rats were anaesthetised with isoflurane (set on 2–3, O2: 400, N2O: 1000). For post-surgery analgesia, Temgesic (1ml in 9 l NaCl, dose: 0.2 ml s.c.) or Metacam (2 mg/ml, dose: 1 mg/kg s.c) was administered.

Sacrifice was 16 wks post-surgery (~ 30w old). Animals were euthanized (at end of the study, or for welfare reasons) by an intraperitoneal overdose of Pentobarbital (200 mg/kg).

Five rats were prematurely sacrificed due to poor clinical condition (DA: sham x1, OVX x2 in the 2–3 days post-surgery due to drawling, lost sutures/weight and/or pneumonia; VRA4: sham x2, approximately three months after surgery due to skin infections). At end-of-study the four groups were: DA-sham (n = 4); DA-OVX (n = 8); VRA4-sham (n = 2); and VRA4-OVX (n = 10).

Animals were weighed on Day 0, 3 weeks and at sacrifice. Urine was collected (at 3 and 16 weeks). At sacrifice 16 weeks post-surgery, left and right femurs were excised and blood sampled via cardiac puncture.

Femurs (R) were scanned (with a slight repositioning of bones between scans) and total femoral bone mineral density (BMD) and bone mineral content (BMC) were measured blinded (PIXI DXA Densitometer and PIXIMus software v2.0, Lunar Corporation, USA). Results are the mean from two scans.

Femurs (L) were scanned using a XUHR 3D-µCT system (MILabs, The Netherlands) by a single operator. Triplicate measurements were performed; re-positioning the region of interest. Image acquisition: peak voltage (50kVp); current (0.21mA); aluminum filter (400 μm); exposure (75ms/projection); circular scan (360^o^ rotation; 0.375^o^ step-angle; 960 projections/rotation). Images were reconstructed with voxel sizes 30 microns (bone density) or 15 microns (histomorphometric measurements).

Total and mid-shaft *femoral bone volume (mm*^*3*^*) and density* (HU) were measured (VivoQuant 3.0 software). Morphometric measurements used Fiji ImageJ (1.52) software and the BoneJ (1.x) plugin [14, 15]. *Cortical* parameters measured in femoral shaft included cross-sectional area (CSA, mm^2^); thickness (mm); periosteal perimeter (mm); minimum, maximum moment of inertia (Imin, Imax (mm^4^). *Trabecular* parameters measured in the distal femoral metaphysis included: trabecular thickness (mm) and spacing (mm); bone volume relative to total volume: connectivity density (1/mm^3^); degree of anisotropy. Results are the mean from triplicate measurements.

Biomechanical properties were evaluated in the (R) femur (Electron E1000 scanner, Instron, UK). *Shear test*: Femur was fixed vertically and an anvil lowered onto the femoral head until femoral neck fracture. *Three-point-bending test*: Femur was placed horizontally on two anvils (15 mm apart) and another anvil lowered onto the midshaft. Load rate (1 mm/min; 250 N). Maximum load (N); load-at-break (N); flexure-extension-at-break (mm); E-modulus (Mpa); energy-at-break (J) were recorded.

*S*erum (CTX-1, osteocalcin, P1NP (ng/ml)) and urine (CTX-1; osteocalcin (mg/mmol) were quantified by ELISA (RatLaps CTX-1; Rat-MID osteocalcin; Rat/Mouse PINP; Immunodiagnostic Systems Ltd. UK). Urine creatinine (mmol/l) quantification used the Vitros 4600/5600 analysis system (Ortho Clinical Diagnostics, USA). For each animal, results are the mean from duplicate measurements (except osteocalcin, for four urine samples). Urinary CTX-1 and osteocalcin are creatinine corrected.

### Statistical analysis

Comparison of weight and percentage weight-gain used 2-way-ANOVA (Sidak’s *post-hoc*). As there was an effect of strain on body-weight from 3 weeks onwards 2-way ANCOVA with body-weight as a covariate and BMD and BMC as independent variables was performed (SAS JMP 16.0).

In the analyses we report the overall effects on bone parameters of rat strain (regardless of OVX status) and from OVX (regardless of strain). Interaction between strain and OVX was also assessed. To assess these overall effects absolute values were compared using 2-way ANOVA (Sidak’s *post-hoc*), or for non-normally distributed data, Kruskal-Wallis test (Dunn’s *post-hoc*) was used.

Changes in bone parameters (delta values) in OVX versus respective sham groups were calculated for each strain (using individual OVX values subtracted from sham means) and reported as percentage change to account for potential differences in baseline levels. When there was an overall effect of OVX (regardless of strain) and/or a significant difference between at least one of the OVX groups compared to respective sham, comparison used unpaired two-way t-test.

Coefficients of variation (CV): BMD/BMC: <2%. Total femur max density ≤ 9%, and < 2% for all other parameters. Morphometry < 9% and < 4% for trabecular and cortical parameters.

Unless otherwise stated, analyses were performed in GraphPad Prism 9. P < 0.05 was considered significant.

## Results

After Day 0 VRA4 rats weighed less than DA although weight-gain was similar in both strains, over time and in response to OVX (supplementary-Table-1, Additional file-1).

**Fig. 1:**
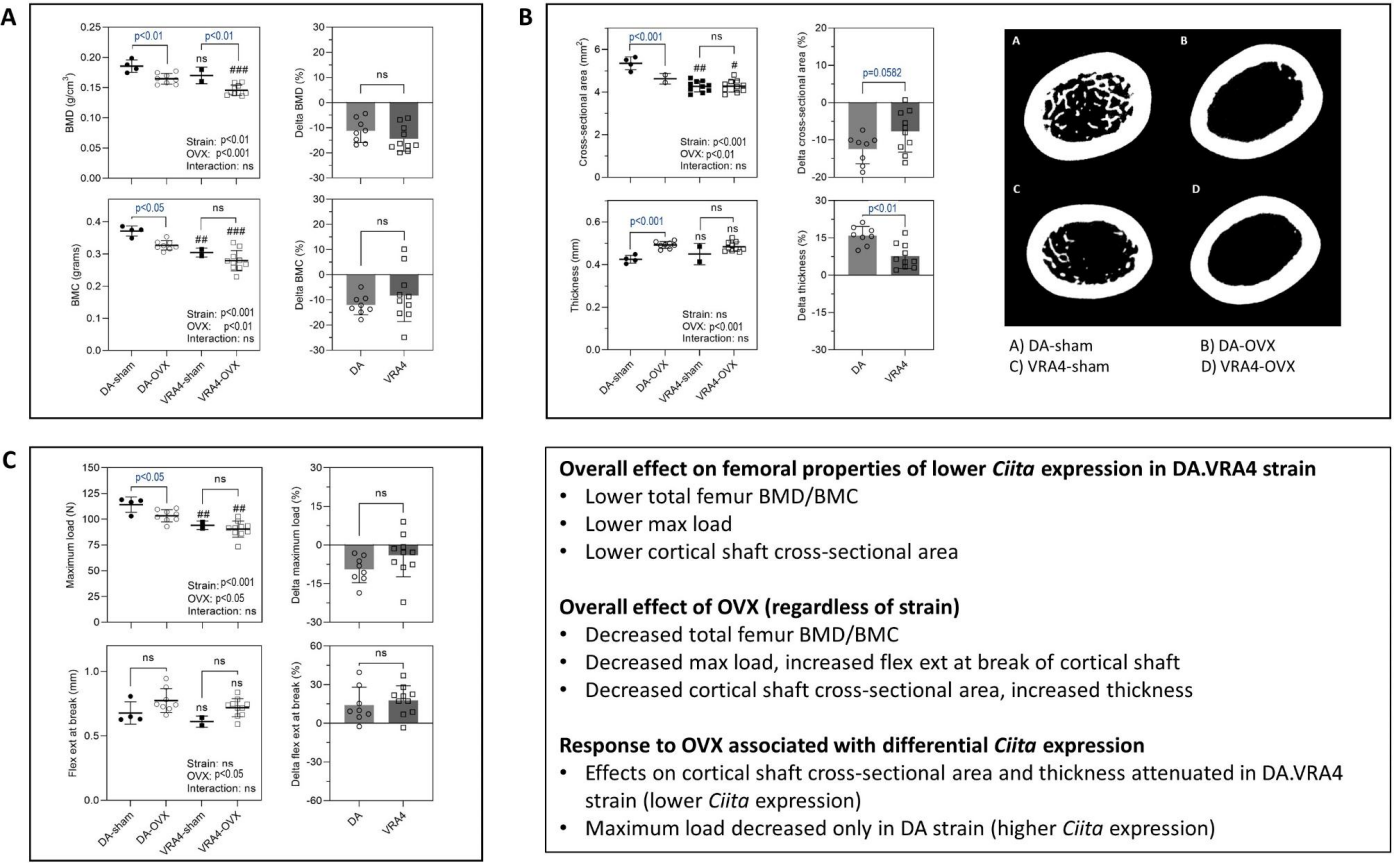
Summary of femoral bone properties associated with differential expression of ***Ciita*** and response to OVX. **Panel A**. Absolute BMD and BMC values (left) and % change (right). **Panel B.** Cortical shaft cross-sectional area and thickness (left), absolute values and % change (middle) and representative images of transverse section of the femoral shaft (right) **Panel C**. Maximum load and flexure extension at break of femoral shaft (left) and % change (right). Comparison with corresponding DA (sham/OVX) ^#^p < 0.05, ^##^p < 0.01, ^###^p < 0.001, ns, not significant

Key results relating to differential *Ciita* expression and bone structural integrity and strength parameters are summarised in Figure-1.

The DA.VRA4 strain had **lower** BMD (p < 0.01) and BMC (p < 0.001) *regardless of OVX status*. BMC was also lower than the corresponding DA groups (p < 0.001–0.01) (**Figure-1 A**; supplementary-Fig-2 A, Additional−file 2 ). Following OVX, there was a decrease in BMD and BMC (supplementary-Fig-1 A, Additional−file 2); but did not differ between strains (supplementary-Fig-1B, Additional−file 2).

Overall, *bone density* and *bone volume* were unaffected by strain and OVX (supplementary-Fig-2,Additional−file 3). Regardless of OVX, *cortical* CSA and Imax levels were lower in the DA.VRA4 strain (p < 0.001 and p < 0.05), whereas periosteal perimeter, thickness, and Imin did not differ between strains (Figure-1B; supplementary-Fig-3 A,Additional−file 4). OVX decreased cortical CSA (across strains), but nominally less in the VRA4 strain (p = 0.058). Furthermore, while OVX increased cortical thickness, this was less pronounced in the DA.VRA4 strain, and no other parameters were affected (supplementary-Fig-3 A,Additional−file 4). *Trabecular* thickness differed between the four groups (p < 0.05), supplementary-Fig-3B, Additional−file 4). Strain per se did not affect thickness, and while OVX induced a slight increase, there were no differences within each strain. No other trabecular parameters were affected (supplementary-Fig-3, Additional−file 4).

In the DA.VRA4 strain, femoral shaft strength and toughness parameters (maximum load, load-at-break, energy- at-break) were lower than in the DA strain (Figure-1 C; supplementary-Fig-4 A, Additional−file 5), whereas flexure-extension-at-break and E-modulus (elasticity parameters) were unaffected by strain. Although OVX decreased maximum load regardless of strain, it did not differ between strains; only reaching significance in the DA strain at *post-hoc* analysis. Following OVX, there was an apparent increase in flexure-extension-at-break (p < 0.05) and energy- at-break (p < 0.01), but did not differ between strains. OVX did not affect load at break or E-modulus: There were no overall effects of strain (regardless of OVX) or OVX (regardless of strain) on shear test parameters (supplementary-Fig-4B, Additional−file 5).

Neither urinary bone turnover marker differed between *strains.* While OVX generally led to increased U-OC levels (p < 0.01–0.05) (Table 1.**i-ii**) the percentage increase was similar in both strains at 3 weeks, but considerably higher at 16 weeks (157% vs. 48%, p < 0.05) in the DA.VRA4 strain (Table 1.**iv-v**).

**Table 1:** Bone biomarker levels in rat strains differentially expressing the *Ciita* gene

	DA-sham *(n = 4)*	DA-OVX *(n = 7–8)*	VRA4-sham *(n = 2)*	VRA4-OVX *(n = 9–10)*	Comparison - overall effects of strain or OVX			(%) Change following OVX	
					(i) Strain	(ii) OVX	(iii) Interaction	(iv) DA	(v) VRA4
*Week 3*									
**U-CTX-1**	25.5 ± 13.1	32.5 ± 19.4	17.5 ± 8.9	34.0 ± 18.3	ns	ns	ns	NA	NA
**U-OC**	21.1 ± 10.4	55.6 ± 29.7 *(7*^a^*)*	37.1 ± 12.9	100.0 ± 40.5 ^b^ *#	ns	p < 0.01	ns	163 ± 141	169 ± 109
***Week 16***									
**U-CTX-1**	8.5 ± 3.5	9.6 ± 2.4 *(7*^d^*)*	9.6 ± 2.5	12.8 ± 3.2 *(9*^d^*)*	ns	ns	ns	NA	NA
**U-OC**	9.1 ± 5.4	13.6 ± 5.6 *(7*^d^*)*	8.0 ± 1.5	20.6 ± 9.4 *(9*,^*c*,d^)	ns	p < 0.05	ns	48 ± 61	157 ± 117^*^
**S-CTX-1**	18.2 ± 2.6	20.3 ± 7.0	17.4 ± 1.9	19.0 ± 3.4	ns	ns	ns	NA	NA
**S-P1NP**	10.2 ± 1.4	8.1 ± 1.5	9.2 ± 1.3	8.3 ± 2.4	ns	ns	ns	NA	NA
**S-OC**	172.8 ± 24.1	198.1 ± 17.0	283.7 ± 55.6 ^**^	218.5 ± 52.2	NA	NA	p < 0.05	145 ± 10	-23 ± 18^***^

Biomarker values are mean ± SD. Urinary (mg/mmol), serum (ng/ml). Urine CTX-1 and osteocalcin are creatinine corrected. NA, not applicable. Ns, not significant

^a^Insufficient sample volume for quantification in one animal. Single-measurement for ^b^one or ^c^three samples (insufficient volume for double-measurements). ^d^Insufficient sample volume to quantify creatinine in one animal

For U-OC, values < LLOQ were set to nominal LLOQ (57.3 ng/ml) for: Week 3, 1x sample each in DA-sham, DA-OVX, and VRA4-OVX; Week 16, all samples in DA-sham, 1x sample each in VRA4-sham and DA-OVX, and 3x samples in VRA4-OVX.

For U-OC, values > ULOQ were set to nominal ULOQ (959 ng/ml) for: Week 3, 1x sample each in VRA4-sham and VRA4-OVX groups

For comparisons with corresponding DA-group *P < 0.05, **P < 0.01. For comparisons with VRA4-sham ^#^ P < 0.05

See also Supplementary Figs. 1–4

An interaction between strain and OVX on serum OC was observed (Table 1.**iv-v**) with higher levels in the DA strain following OVX, while decreasing in DA.VRA4.

## Discussion

This study investigated bone properties in rat strains with allelic differences in expression of the *Ciita* gene, encoding the pro-inflammatory protein Ciita. While the results should be interpreted with caution, differential Ciita expression may be associated with changes to structural integrity and strength with the low Ciita expressing strain (DA.VRA4) displaying lower BMD, BMC and cross-sectional area. Other mechanical properties reflecting bone strength/stiffness were also affected, such that lower energy was required to fracture. Strain differences may also explain differences observed following OVX.

In mice *Ciita* overexpression leads to an osteopenic phenotype [6], and indirect induction of *Ciita* expression induces bone loss [2]. In post-menopausal women, lower *CIITA* expression associated with increased bone mass [7]. However, in this experimental model lower Ciita levels appeared to be overall detrimental to bone.

Without having performed histomorphometry we can only speculate whether this observation is due to sample size or reflects the complex negative and positive feedback loops regulating bone homeostasis. In line with our findings, in vitro *Ciita *over expression in murine osteoclasts (responsible for bone resorption) decreased osteoclastogenesis, whereas down-regulation (siRNA) enhanced it [16].

The observations from the present study collectively, albeit tentatively, suggest that Ciita levels may have a role in the skeletal degradation induced by estrogen-deficiency. Estrogen is proposedly important in regulating Ciita [17, 18]. In vitro studies demonstrate estradiol down-regulates Ciita expression in breast carcinoma cell lines (reversed by anti-estrogens) [18] and indirectly down-regulates Ciita expression in macrophages [17]. We can speculate that in our model, OVX increased Ciita levels in both strains, since the suppressing effect of estrogen was eliminated which is in keeping with mice studies, where OVX increased *Ciita* expression, leading to bone loss [2]. Ovariectomy was accompanied by decreased BMD and BMC, but the extent did not appear to be affected by differences in *Ciita* expression. Interpreting the effects on bone strength is hampered by the sham group sizes. The lower *Ciita* expression in DA.VRA4 rats may have delayed or reduced negative effects of OVX on bone mass, but it is possible the regulatory mechanisms differ in early and late post-menopause. Also, our knowledge of gene-environment and gene-gene interactions involved in regulating Ciita are limited.

Although using a rat strain with congenitally low *Ciita* expression should more closely represent the natural differences in genetic background in humans, compared to knock-out models, the study has limitations making it difficult to do more than tentatively suggest that differential Ciita expression is accompanied by altered bone properties.

## Limitations

The model does not fully translate to post-menopausal osteoporosis and the variation in age at OVX (12–17 weeks) makes it difficult to distinguish between effects on bone acquisition and bone loss, even if an inflammatory environment affects bone metabolism. On the other hand, in intact mice overexpressing Ciita, bone loss has been observed at 24 weeks, but not at 6 or 12 [8]. Also, the success of OVX was not verified, by uterine weight or estrogen assays.

The low size of the sham groups (premature termination due to poor clinical condition) is a major limitation, not fully offset by the 2 × 2 study design (maximising power within this setting) and the very low standard deviation of many parameters which enabled *post hoc* identification of apparent differences. Replacing the lost animals would have ensured more robust results.

Gene or protein expression levels of Ciita were not measured, although differential expression of *Ciita* has been well-characterised in these strains, and we assume bone to be in line with other tissues. Genetic drift or other genetic differences in the two rat models may be possible.

Only femoral properties were assessed; compression tests on lumbar vertebrae could add information on trabecular bone strength, possibly clarifying why changes were predominantly observed in cortical but not trabecular bone. Additional structural variables, might have helped interpretation, as could in situ cell number and activity.

These acknowledged limitations may explain several paradoxical results. In DA rats cortical thickness increased, but CSA decreased, despite an unchanged periosteal perimeter and appears to conflict with the decreased bone strength observed. Additionally, although osteocalcin usually decreases following OVX, urinary levels increased.

## Conclusion

The study tentatively suggests association between Ciita expression and bone structural and mechanical properties, and possibly response to estrogen deficiency, overall supporting a role for inflammation influencing skeletal integrity. This highly interesting pathophysiological pathway warrants exploring in relation to osteoporosis and even bone accrual.

### Electronic supplementary material

Below is the link to the electronic supplementary material.

Supplementary Material 1

Supplementary Material 2

Supplementary Material 3

Supplementary Material 4

Supplementary Material 5

## Data Availability

All data generated or analysed during this study are available from the corresponding author upon reasonable request.

## Abbreviations

BMC: Bone Mineral
BMD: Bone Mineral Density
CIITA: Class II transactivator
CSA: Cross-sectional area
CTX-1: Serum C-telopeptide cross-link type 1 collagen
DA: Dark Agouti
OC: Osteocalcin
OVX: Ovariectomy
P1NP: Propeptide of type 1 procollagen
µCT: Micro computed tomography
