# Supplementary material for: Differential expression of the inflammatory ciita gene may be accompanied by altered bone properties in intact sex steroid-deficient female rats
Source: BMC Res Notes. 2023 Dec 19;16:372. doi: 10.1186/s13104-023-06543-4 (PMC10729448; doi:10.1186/s13104-023-06543-4)
Supplement: Supplementary file 1 — Supplementary Material 1 [file 13104_2023_6543_MOESM1_ESM.pdf]

Supplementary Table 1. Body-weight (g) in rat strains differentially expressing the *Ciita* gene

	Normal-to-high *Ciita* expression		Lower *Ciita* expression		Comparison for overall effects		
	**DA-sham** *(n=4)*	**DA-OVX** *(n=8)*	**VRA4-sham** *(n=2)*	**VRA4-OVX** *(n=10)*	**Strain**	**OVX**	**Interaction**
**Day 0** [a]	175±10 *(15)*		167±12 *(14)*		-	-	**-**
**Day 0** [b]	173 ± 8 *(5)*	176 ± 11 *(10)*	163 ± 16 *(4)*	168 ± 11 *(10)*	ns	ns	ns
**Week 3**	188 ± 8 *(4)*	213 ± 10 *(8)***	175 ± 12 *(4)*	197 ± 10 *(10)**#*	p<0.001	p<0.01	ns
**Week 16**	207 ± 2 *(4)*	222 ± 12 *(8)**	187 ± 17 *(2)*	211 ± 8 *(9)***	p<0.001	p<0.01	ns
**Δ Weight** (D0 - w16)	18% *(4)*	27% *(8)**	20% *(2)*	28% *(9)*	ns	p<0.001	ns

At designated Day 0, rats were approximately 14 weeks (range 12-17). Animals sacrificed 16 weeks after surgery (approximately 30 weeks old). Weight (g) reported as mean ± SD. [a)] Before allocation into surgical group. [b)] After allocation.

Day 0[a]: Unpaired two-tailed t-test between strains. Day 0[b], Weeks 3, 16: 2-way ANOVA (*post hoc* Sidak's multiple comparisons test) between groups.

For comparisons within each strain *p<0.05 and **p<0.01.

For comparisons with group DA-OVX [#]p<0.05.
