# Supplementary material for: Differential expression of the inflammatory ciita gene may be accompanied by altered bone properties in intact sex steroid-deficient female rats
Source: BMC Res Notes. 2023 Dec 19;16:372. doi: 10.1186/s13104-023-06543-4 (PMC10729448; doi:10.1186/s13104-023-06543-4)
Supplement: Supplementary file 2 — Supplementary Material 2 [file 13104_2023_6543_MOESM2_ESM.pdf]

Supplementary Figure 3

A) Cortical and B) trabecular morphometric measurements in the experimental groups and strain associated OVX change (femur)

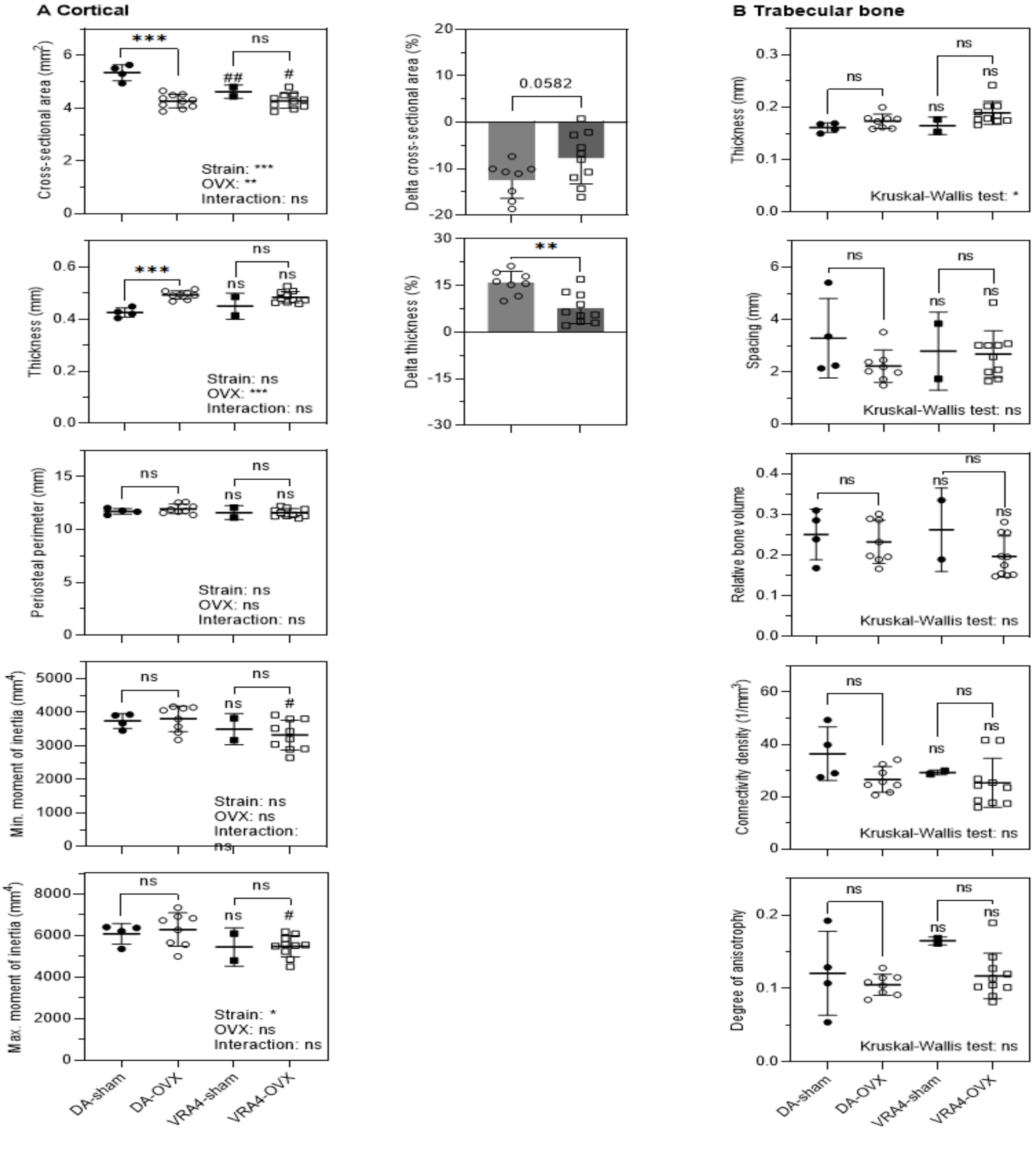

Panel A: Absolute and delta values in femoral shaft. **Panel B**: Absolute values in femoral metaphysis.

Values are individual means ± SD. DA-sham (n=4), DA-OVX (n=8), VRA4-sham (n=2), VRA4-OVX (n=10). DA (n=8), VRA4 (n=10).

Cortical results: 2-way-ANOVA (*post hoc* Sidak's multiple comparisons test). Delta values compared using unpaired two-way t-test.

Trabecular results: Kruskal-Wallis test (*post hoc* Dunn's multiple comparisons test).

*p<0.05, **p<0.01, ***p<0.001. Comparisons with corresponding DA group (sham/OVX) $^{\#}$p<0.05, $^{\#\#}$p<0.01, $^{\#\#\#}$p<0.001, ns, not significant.
