# Supplementary material for: Differential expression of the inflammatory ciita gene may be accompanied by altered bone properties in intact sex steroid-deficient female rats
Source: BMC Res Notes. 2023 Dec 19;16:372. doi: 10.1186/s13104-023-06543-4 (PMC10729448; doi:10.1186/s13104-023-06543-4)
Supplement: Supplementary file 3 — Supplementary Material 3 [file 13104_2023_6543_MOESM3_ESM.pdf]

Supplementary file-Figure 2.

Bone volume, maximum and mean bone density in A) the experimental groups and B) strain associated OVX change (femur)

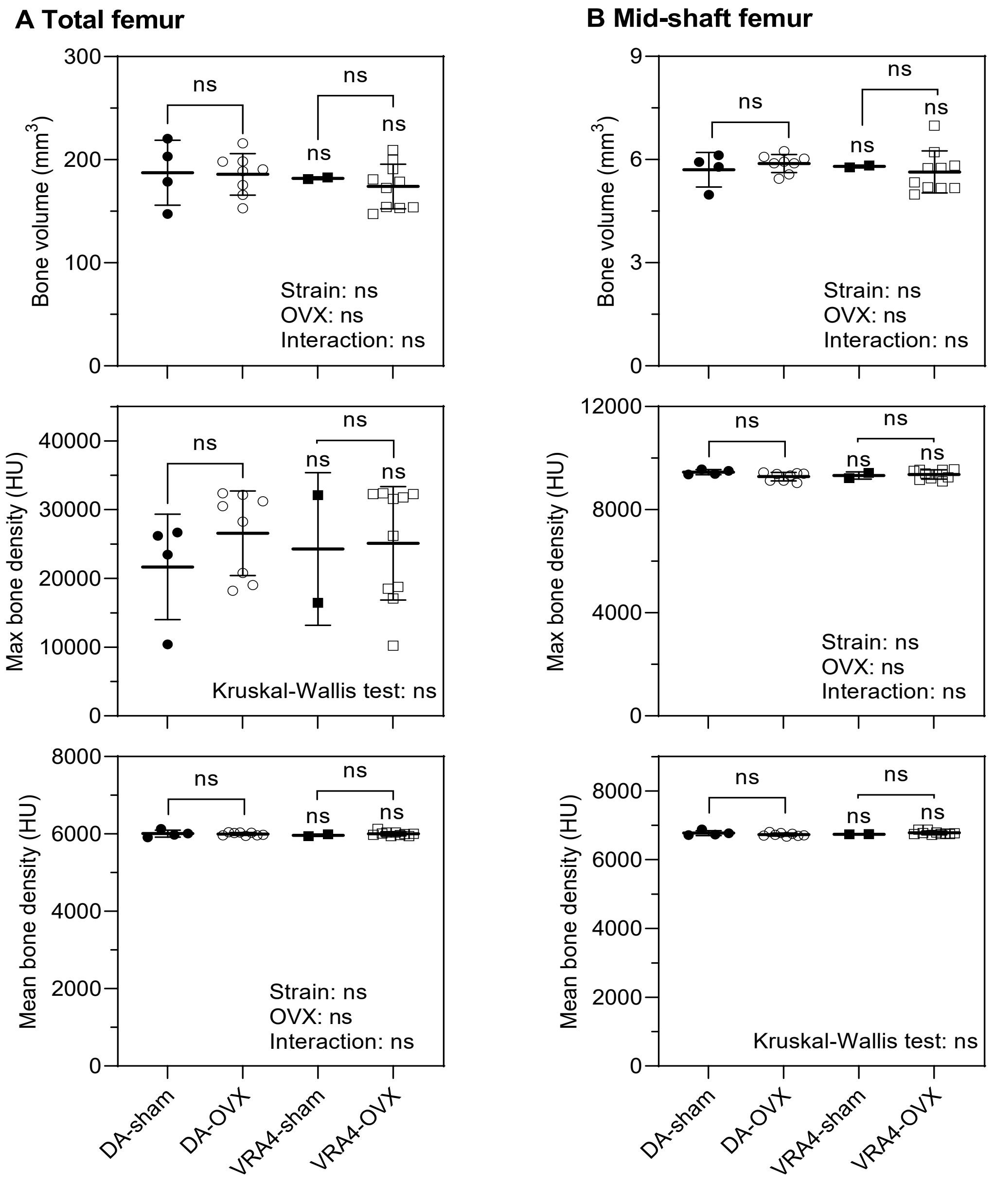

Panel A: Total femur. **Panel B**: Mid-shaft femur. DA-sham (n=4), DA-OVX (n=8), VRA4-sham (n=2), VRA4-OVX (n=10). Values are individual means ± SD. Comparisons use 2-way ANOVA (*post hoc* Sidak's multiple comparisons test) or Kruskal-Wallis (*post hoc* Dunn's multiple comparisons test).
