# Supplementary material for: Differential expression of the inflammatory ciita gene may be accompanied by altered bone properties in intact sex steroid-deficient female rats
Source: BMC Res Notes. 2023 Dec 19;16:372. doi: 10.1186/s13104-023-06543-4 (PMC10729448; doi:10.1186/s13104-023-06543-4)
Supplement: Supplementary file 4 — Supplementary Material 4 [file 13104_2023_6543_MOESM4_ESM.pdf]

Suppl. Figure 4. Biomechanical properties of A) femoral shaft and B) femoral neck in the experimental groups and strain associated OVX change

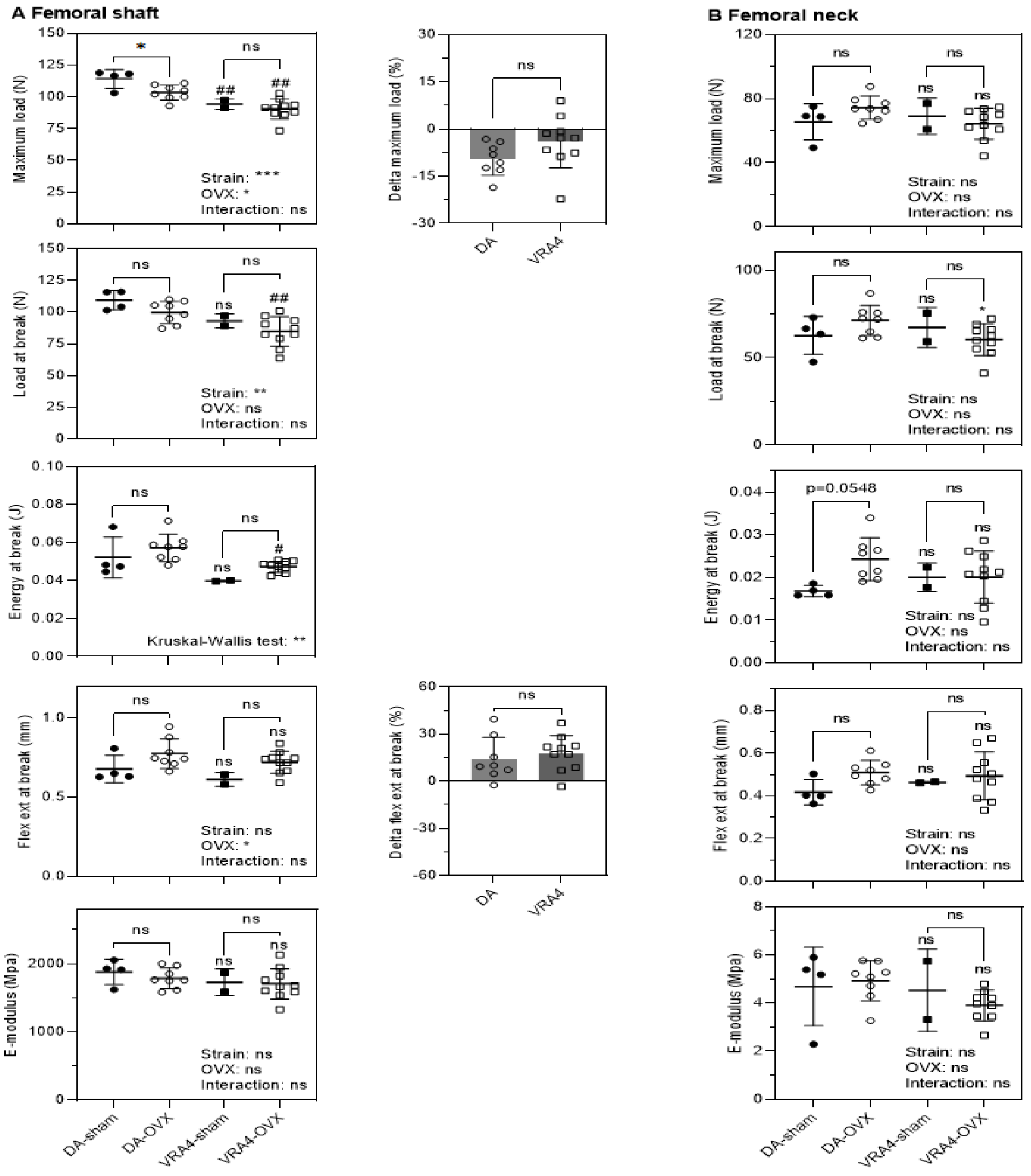

Panel A: Femoral shaft absolute and delta values (3-point-bending test). **Panel B**: Femoral neck absolute values (shear test). DA-sham (n=4), DA-OVX (n=8), VRA4-sham (n=2), VRA4-OVX (n=10) (except for femoral neck E-modulus where VRA4-OVX (n=9), DA (n=8), VRA4 (n=10).

Values are individual means ± SD. Comparisons use 2-way-ANOVA (*post hoc* Sidak's multiple comparisons test) for absolute values unless otherwise stated or Kruskal-Wallis test (*post hoc* Dunn's multiple comparisons test). Delta values compared by unpaired two-way t-test. *p<0.05, **p<0.01, ***p<0.001. Comparisons with corresponding DA group (sham/OVX) #p<0.05, ##p<0.01, ###p<0.001, ns, not significant.
