# Supplementary material for: Differential expression of the inflammatory ciita gene may be accompanied by altered bone properties in intact sex steroid-deficient female rats
Source: BMC Res Notes. 2023 Dec 19;16:372. doi: 10.1186/s13104-023-06543-4 (PMC10729448; doi:10.1186/s13104-023-06543-4)
Supplement: Supplementary file 5 — Supplementary Material 5 [file 13104_2023_6543_MOESM5_ESM.pdf]

Supplementary Figure 1.

DXA measured BMD and BMC in A) the experimental groups and B) strain associated OVX change

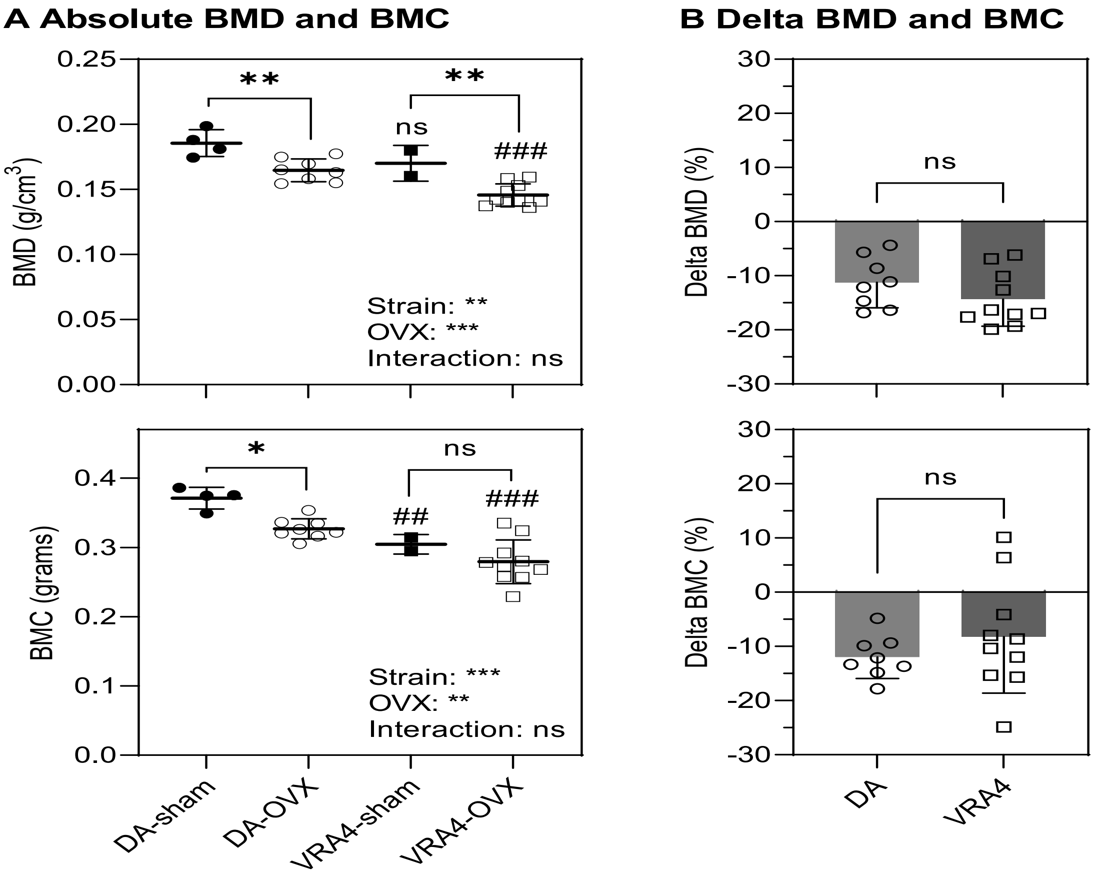

Panel A: Total femur **a**bsolute BMD and BMC measured at 16 weeks in the four experimental groups. DA-sham (n=4), DA-OVX (n=8), VRA4-sham (n=2), VRA4-OVX (n=10). Compared using 2-way-ANOVA, with *post hoc* Sidak's multiple comparisons test.

Panel B: Change in BMD and BMC (delta-values) after OVX in the two strains. DA-OVX (n=8), VRA4-OVX (n=10). Compared using unpaired two-way t-test.

Values are individual means ± SD. $^{*}p<0.05$, $^{**}p<0.01$, $^{***}p<0.001$. Comparisons with corresponding DA group (sham/OVX) $^{\#}p<0.05$, $^{\#\#}p<0.01$, $^{\#\#\#}p<0.001$, ns, not significant.
